# Caregivers’ role regarding managing postoperative pain of hospitalised children (0–3 years)

**DOI:** 10.4102/hsag.v30i0.2739

**Published:** 2025-03-11

**Authors:** Sylvia Oger Ofosu-Dwamena, Carin Maree, Seugnette Rossouw, Varshika Bhana-Pema

**Affiliations:** 1 Department of Nursing Science, Faculty of Health Sciences, University of Pretoria, Pretoria, South Africa

**Keywords:** caregiver, hospitalisation, postoperative pain management, nurses, children (0–3 years)

## Abstract

**Background:**

Acute postoperative pain is a common surgical symptom affecting 40% - 80% of patients. Postoperative pain produces much distress in children. Effective postoperative pain management is a human right. Various stakeholders, including caregivers, are involved in the management of children’s postoperative pain. However, the role of the caregiver is accentuated during the child’s discharge, with limited studies focusing on the role during hospitalisation.

**Aim:**

This study aimed to describe how caregivers manage their children’s (0–3 years) postoperative pain during hospitalisation and explore caregivers’ expectations about how their hospitalised children’s postoperative pain is managed.

**Setting:**

The study setting was a regional hospital in Ghana.

**Methods:**

The research employed a descriptive qualitative methodology. Purposive sampling was used to recruit caregivers. Data were collected using individual in-depth interviews. The transcripts were thematically analysed using Clarke and Braun’s framework.

**Results:**

The researchers identified four themes: caregiver’s experiences of caring for children with postoperative pain, caregivers’ assessment of children’s postoperative pain, caregivers’ assistance with management and expectations of the caregivers concerning the management of postoperative pain in their hospitalised children (0–3 years).

**Conclusion:**

Caregivers in this study acknowledged the fact that the postoperative pain experienced by their children had a noteworthy emotional effect on them.

**Contribution:**

Nurses must adequately educate caregivers on managing postoperative pain in hospitalised children (0–3 years). Again, the nurses must be with the child and the caregiver, as this enhances collaboration and adequate postoperative pain management in these children.

## Introduction

Acute postoperative pain is the presence of pain or discomfort following surgical interventions (Bakir et al. 2021). It is a common surgical symptom affecting 40% – 80% of patients (Skervin & Levy 2020). The postoperative period, which is the period after surgery, produces much distress in children. Despite ongoing attempts to enhance postoperative pain management in children, a significant proportion of children continue to experience ineffective management of postoperative pain (Vittinghoff et al. 2018). Ineffective pain management among children has dire consequences for the growing child. These consequences are impaired respiratory system function, sleep disturbance, poor fluid intake and suppressed immunity (Bakir et al. 2021; Kasahun, Sendekie & Abebe 2023). Additionally, ineffective postoperative pain management can lead to chronic pain, which is pain that persists or has a duration beyond 3 months (Dugan et al. 2022). Managing postoperative pain for children effectively is a human right (Brennan, Lohman & Gwyther 2019).

Effective management of postoperative pain involves pain assessment and pain treatment. Pain assessment detects pain intensity, quality, location and duration (Trottier et al. 2022). The treatment of postoperative pain in children encompasses the implementation of both the use of medications (pharmacological) and non-pharmacological strategies. Non-pharmacological strategies include music, sucking, breastfeeding and videos, among others (Short, Pace & Birnbaum 2017).

Studies have reported that children are most likely to suffer from ineffective pain management (Crellin et al. 2018; Wren et al. 2019), and vulnerable among them are neonates and infants (Wren et al. 2019). Additionally, for children (0–3 years) who cannot verbalise if they experience pain because of their developmental stage, self-reporting, which is the ideal way of assessing pain (Crellin et al. 2018), cannot be utilised. Hence, an alternative method of assessment is needed for their postoperative pain management.

Postoperative pain management for children aged birth to 3 years involves anaesthetists, nurses, paediatricians and caregivers, with the caregiver’s crucial role inevitable. The caregiver plays an important role in the postoperative pain management of children during hospitalisation and also needs to continue with the pain management after discharge. But little is known about their role and experiences during hospitalisation. Best practice guidelines recommend involving the caregiver in managing postoperative pain in children to enhance its effectiveness (Kaki et al. 2021; Vittinghoff et al. 2018). Caregivers play a crucial role in managing children’s pain, especially when considering psychosocial factors, as they understand their children’s preferences and dislikes (Bennett 2019). Nurses are expected to help caregivers assist in managing postoperative pain during hospitalisation, emphasising the importance of non-pharmacological measures (Bennett 2019). However, the role of the caregiver is accentuated during the child’s discharge. Most studies focus on postoperative management of children after discharge at home (Dagg et al. 2020; Saigh & Saigh 2023). The information on caregivers’ involvement in the postoperative pain management of children during hospitalisation is limited. Much is written about the postoperative pain of children, but little is known about the caregiver’s role and experiences thereof. This is also the case in Ghana.

Also in Ghana, most studies on postoperative pain management focus on nurses, with limited studies on the caregiver (Kusi Amponsah et al. 2019; Ofosu Dwamena, Druye & Asamoah Ampofo 2020, 2021). In Ghana, several procedures are frequently conducted on children, including umbilical hernia repair, inguinal herniotomy, circumcision, removal of lumps, cleft lip repair and orchidopexy (Amponsah & Etwire 2018; Kuorsoh 2021). Children between the ages of 1 and 5 years account for 52% of these procedures (Amponsah & Etwire 2018). Postoperative pain remains a significant complication in this context, especially in children, and caregivers play a crucial role in managing their children’s pain. This study aimed to describe how caregivers manage children’s (0–3 years) postoperative pain during hospitalisation and explore caregivers’ expectations about how their hospitalised children’s postoperative pain is managed.

## Research methods and design

### Study design

This study utilised a descriptive qualitative design. This strategy was employed to obtain an in-depth understanding of the caregivers’ experiences of their management of postoperative pain in their hospitalised children, through direct interaction with the caregivers.

### Study setting

The study setting was a regional and referral hospital in the Sekondi-Takoradi Metropolis of the Western region of Ghana. The hospital, established in 1938, has two operating theatres. There are two surgical wards: male and female surgical wards. There is no surgical ward for children after surgery; hence, after any surgery, children are nursed in the recovery ward, after which they are sent to the female surgical ward when they are stable. The caregivers stay with their children throughout their hospitalisation. The researcher chose to conduct the study because this facility served as a referral hospital for two administrative regions out of the 16 regions in Ghana. Also, approximately 300 paediatric surgeries are conducted each year in this hospital.

### Population and sampling

The study population consisted of caregivers who cared for their children after surgery at a regional hospital. As operationalised, the caregiver in this study is ‘any individual, whether a mother, father or third party who takes active care of the child after surgery’. Purposive sampling was employed to recruit caregivers as the researchers aimed to find participants with extensive experience with the investigated phenomenon.

#### Inclusion criteria

The inclusion criteria for the caregivers were as follows: (1) a caregiver taking part in the care of the child at the hospital, (2) a caregiver who has spent at least 3 days with the child in the hospital and (3) a caregiver willing to participate in individual face-to-face interviews with the researcher.

#### Exclusion criteria

The study excluded: (1) caregivers whose children underwent minor surgery and were hospitalised for less than 3 days and (2) caregivers who were unwilling to take part in the study. The sample size was planned for 15 participants or until data saturation was achieved. Data saturation occurs when new insights are not identified, and data repetition occurs, making further collection unnecessary, indicating a sufficient sample (Hennink & Kaiser 2021). The principal researcher found that by the interview of the 12th participant, no new information emerged so she conducted three additional interviews to confirm this.

### Data collection

Pilot testing was done with four caregivers with children who had undergone surgery at another hospital to determine the suitability of the interview guide and to provide the principal researcher with experience in conducting the interview. The principal researcher obtained access to the female surgical ward in charge after she received ethical clearance and a letter of permission. She was introduced to the nurses in the ward and had an opportunity to brief them about the purpose of the study, and the inclusion and exclusion criteria of the participants. The nurses agreed to help with the recruitment of the participants in the female surgical ward. After recruiting the participants, the researcher received informed consent to conduct the interviews and to audio record the interview.

Data were collected by the principal researcher face-to-face in a separate room in the ward to ensure privacy. The interview was scheduled with the caregiver on the third day after the child’s surgery. Data were collected using individual in-depth interviews with a semi-structured interview guide. The two broad questions that were asked during the interview were: (1) How do you assist in managing the postoperative pain of your child during hospitalisation? (2) How do you expect your child’s postoperative pain to be managed during hospitalisation? Probes were used to give details of the questions and field notes were taken. Some interviews were conducted in English, while others were conducted in Fante, a local Ghanaian language. Those interviews conducted in Fante were translated into English before transcribing. The data were collected from June to August 2023. The average length of each interview was 45 min. The interviews were audio-recorded with the permission of the participants.

### Data analysis

The principal investigator transcribed the audio recording from the individual in-depth interview verbatim. The transcripts were analysed using the thematic analysis framework presented by Clarke and Braun (2013) to describe themes and subthemes. This type of analysis involves six steps: (1) familiarisation of data, (2) generating initial codes, (3) searching of themes, (4) reviewing themes, (5) defining and naming themes and (6) finalising the analysis. In the first stage (familiarisation of data), the raw data were read multiple times to enhance it. The second step involved creating initial codes. The researchers generated the initial codes from the data and arranged them into themes. During the third step, which involved searching for themes, the transcripts were coded numerically per the themes found. The first codes formed a list of potential themes and subthemes. In the fourth step, reviewing themes, the researchers considered whether the themes reflected the underlying codes. The researcher proceeded to combine or exclude codes that either conveyed a similar narrative or, following reflection, were deemed irrelevant to the research objective. As a result, it was suitable for certain codes to create subthemes to further categorise the content of the main themes. In the fifth step, the researchers named and defined the themes. Using the research objectives, the researchers assessed how each theme fits into the data’s overall story. The final step involved finalising the analysis and preparing it for presentation.

### Trustworthiness

The researchers used credibility, dependability, confirmability, transferability and authenticity, as proposed by Guba and Lincoln (1989), to ensure the rigour or trustworthiness of the study. To achieve credibility, the researchers ensured the following: prolonged participant engagement during data collection, data saturation, member checking and literature control. With member checking, the researchers returned to the participants to check whether the data collected accurately described the experiences of caregivers managing postoperative pain among children. Some caregivers were still on admission during the member checking, while some were contacted when they brought their children for review after discharge. Dependability was enhanced through a detailed description of the methodology to make replication possible should anyone be interested. Confirmability was enhanced by using direct quotes to demonstrate actual data. To ensure transferability, the researchers made an effort to provide specifics about the study’s setting and the procedures followed throughout data collection and analysis. Authenticity was made possible through the verbatim transcription of the interview.

### Ethical consideration

Research Ethics Committee of the Faculty of Health Sciences, University of Pretoria, approved this study (Reference no.: 708/2022). The researcher adhered to all ethical principles of research. The researcher acquired written informed consent from the participants. The participants had the freedom of choice to participate in the study and were not coerced. They were also made to understand that they were free to withdraw from the study without any penalty or consequence on their child’s management. After agreeing to be part of the study, the researcher also received approval from the participants to audio record the interview. The participants did not receive any remuneration for the study.

## Results

### Demographic characteristics of the participants

Fifteen caregivers of children who had undergone surgery participated in this study. Eight were between the ages of 31 years and 40 years, four were between the ages of 18 years and 30 years and the remaining three were between the ages of 51 and 60 years. The majority of the participants (*n* = 12) were the biological mothers of the children; the rest were grandmothers (*n* = 2) and an aunty (*n* = 1). The educational level of most of the caregivers was tertiary (*n* = 9), followed by secondary (*n* = 3), then principal (*n* = 2), and the remaining one had no formal education. The majority of the children of the caregivers were between the ages of 24 and 36 months (*n* = 8), followed by 12 and 23 months (*n* = 5) and 0 and 11 months (*n* = 2). Most children were males (*n* = 12), while the rest were females (*n* = 3). Out of the 15 participants, 4 of the children of the caregivers underwent hernia repair, and two each underwent appendicectomy and tonsillectomy. The remaining seven children underwent orchidopexy, incision and drainage of abscess, excision of ganglion, contracture release and urethroplasty.

### Themes and subthemes

Table 1 shows the themes and subthemes that were developed from the data through data analysis. The researchers identified four themes and seven subthemes.

**TABLE 1:** Themes and subthemes: Caregivers’ experiences and management of postoperative pain of their hospitalised children.

Themes	Subthemes	Codes
Caregivers’ experiences of caring for children with postoperative pain	Emotional experiences	Anxiety Sorrow Feeling financially burdened
Caregivers’ assessment of postoperative pain of their children	Subjective measures	Verbalisation of pain
	Behavioural measures	Crying Facial expression Irritable behaviour Pointing to the wound Difficulty lying on the affected side Refusal to eat
	Physiological measures	Increased body temperature
Caregivers’ assistance with the management of the postoperative pain of their children	Non-pharmacological treatment	Psychosocial support Touch Watching videos Music Conversation Breastfeeding
Expectations of caregivers concerning the management of their children’s postoperative pain	Caregivers’ expectations of nurses	Administer medications whenever there is pain Be present with the child Give adequate education Children to be hospitalised postoperatively in a children’s ward
	Caregiver’s expectations of the hospital	

#### Theme one: Caregiver’s experiences of caring for children with postoperative pain

**Emotional experiences:** Under the first theme, one subtheme was identified when the caregivers were asked to describe their experiences of caring for their children with postoperative pain: emotional experiences.

The participants indicated that their children’s postoperative pain had an emotional impact on them as caregivers. They experienced anxiety, sorrow and feeling financial burdens when their children had postoperative pain.

The caregivers expressed anxiety about their children’s postoperative pain, using their past experiences to judge the level of pain they might experience. The caregivers shared the following:

‘I was scared and contemplating if my grandson can endure the pain. I experienced ten years ago due to fibroid considering his age.’ (Grandmother, 54 years)

The caregivers expressed sadness, secret crying and devastation upon realising their children were in pain. In this regard, participants talked about their experiences:

‘Hmmm … It was not easy at all. Initially, when I saw my daughter, I really cried but did not let anyone see me.’ (Biological mother, 32 years)

A participant expressed her principal concern, feeling burdened about how she would cover her child’s expenses without any insurance:

‘The money involved gave me sleepless nights, but finally, we were able to make money to finance the surgery.’ (Biological mother, 25 years)

#### Theme two: Caregivers’ assessment of the postoperative pain of their children

When asked how they knew their children were in pain, the caregivers mentioned using subjective, behavioural and physiological measures.

**Subjective measures:** The caregivers mentioned that some of the children who were 3 years old were able to self-report or verbalise their own postoperative pain. From a participant’s experience, her child, who was 3 years old, mentioned that he was feeling pain. The participants’ comments were:

‘He also told me that it was paining him. You know he is three years old and can talk.’ (Biological mother, 30 years)

‘My son will, most of the time, say pain and show me the area. That helps me to know he was experiencing postoperative pain.’ (Biological mother, 25 years)

**Behavioural measures:** The results of this study revealed that caregivers used varied behavioural measures to assess their children’s postoperative pain. These measures included crying, facial expression, irritability, restlessness, pointing to the wound, difficulty lying on the affected side and refusal of food.

Postoperative pain in children was often assessed using intense crying, different from their usual crying, with some caregivers describing it as shouting. A caregiver stated:

‘The nurses told me she had given her medication for pain, but my child cried at night in such a way that he had never done before.’ (Biological mother, 32 years)

Caregivers noted facial expressions like frowning, frowning, squeezing, sorrowful and unusual facial expressions to detect their children’s pain. The participants shared their experiences:

‘After surgery, my son’s cheerful smile disappeared, causing him to look as if he has been offended. However, he returned to his usual self three days later, indicating a relief of pain.’ (Biological mother, 30 years)

Caregivers noted irritable behaviour to detect postoperative pain in children by observing restlessness and irritability, which diminished after pain management. Caregivers describe observing signs of irritable behaviour in their child to assist them in knowing if their child is experiencing pain:

‘I can also say that the pain made her very irritable; she did not want people to get closer, and she did not even want to sleep on the hospital bed, but once she was relieved from pain, she calmed down.’ (Biological mother, 32 years)

‘The way my son was behaving. That is, he could not be still. As if he was restless. At some point, he wanted me to carry out, and at the same time, he wanted to be put down.’ (Biological mother, 46 years)

Caregivers reported their children attempting to touch the wound site and pointing to it, interpreting it as pain. Some of the participants reiterated:

‘He attempted to touch the plaster, but when restrained, he pointed at the wound, indicating the exact spot of pain.’ (Aunty, 40 years)

Caregivers confirmed their children’s postoperative pain as they could not lie on the affected side. A caregiver gave an account as follows:

‘From the theatre, she could not lie on her tummy but now can and even roll in bed without squeezing her face.’ (Grandmother, 58 years)

The caregivers interpreted the children’s refusal of food as a sign of pain, using it as a pain assessment measure. A participant shared:

‘My child experienced severe pains about 4/5 and was refusing food. It is only today (4th day postoperative) that he has eaten well.’ (Biological mother, 26 years)

**Physiological measures:** The caregivers utilised increased body temperature as the sole physiological measure for evaluating postoperative pain.

According to the caregivers, postoperative pain made their children’s temperature rise above normal. These are accounts of caregivers:

‘His pain increased his temperature, and I noticed he was warm upon touch. The nurse confirmed this with a thermometer.’ (Grandmother, 58 years)

‘My daughter’s temperature was high and that made me know she was in pain.’ (Biological mother, 46 years)

#### Theme three: Caregivers’ assistance with management of the postoperative pain of their children

**Non-pharmacological treatment:** The study found that postoperative pain in children was reduced using non-pharmacological measures such as psychological support, touch, video, music, conversation and breastfeeding.

The psychosocial support caregivers gave included their presence, reassurance, and walking and carrying the child. The caregiver’s presence helped reduce the postoperative pain of the children by calming them. A caregiver accounted:

‘I stayed with him throughout because I realised that he did not want anyone to get closer to him. I mean the nurses and the doctors.’ (Biological mother, 30 years)

Participants reported that they used reassuring words to treat their children’s postoperative pain:

‘I assured him that everything would be fine and that we would be discharged home for him to see his baby sister.’ (Aunty, 40 years)

Caregivers used psychological support by walking and carrying the child to distract children from postoperative pain. A caregiver said:

‘Pacing up and down the staircase carrying the child distracted him from the pain on the second day after surgery and this helped the child to fall asleep.’ (Biological mother, 35 years)

Participants in this study utilised touch to assist in managing their children’s postoperative pain, such as cuddling and rubbing of hands. Caregivers shared:

‘After the first day, I could pick her up and cuddle her in my arms, which greatly calmed her down.’ (Biological mother, 32 years)

‘I was rubbing my palm at his back and head.’ (Grandmother, 58 years)

The participants reported that postoperative children were managed with music through phone or caregiver singing. A participant said:

‘I sang for him, taking his mind off the pain. He initially disliked it, but over time, he started playing music from his phone.’ (Biological mother, 22 years)

Participants utilised videos, mainly cartoons, on caregivers’ phones to distract children who experienced postoperative pain. A participant shared:

‘I used my phone to distract my crying daughter by playing cartoons, which helped distract her from the pain.’ (Biological mother, 35 years)

Caregivers employed techniques such as conversation to alleviate their children’s postoperative pain. A participant mentioned:

‘I engaged him in a lot of talking that it we conversed together mostly reminding him his siblings were waiting for him to get home.’ (Biological mother, 28 years)

A caregiver mentioned that she breastfed her son, and that was useful in managing his postoperative pain as directed by the nurses:

‘I was also informed by the nurses to breastfeed my son which I did and saw it was helpful.’ (Biological mother, 30 years)

#### Theme four: Expectations of caregivers concerning the management of their children’s postoperative pain

Theme four produced two subthemes when the caregivers were asked about their expectations concerning their children’s postoperative pain management, namely expectations from nurses and expectations from the hospital.

**Expectations from nurses:** Nurses were expected to administer pain medication, be present with the child and provide education.

A caregiver mentioned that she expected the nurses to give medications to the child whenever the child was experiencing postoperative pain. To her, it should be an injection because it works faster. A participant said:

‘I was expecting the nurses to give him injections because I know injections work faster.’ (Biological mother, 38 years)

Caregivers anticipated nurses to spend more time with the child even if caregivers were present:

‘I expected the nurses to be with the child for some time because I lack knowledge about how to care for the child.’ (Aunty, 40 years)

The caregivers in this study expected the nurses to give adequate information, but that was not the case. A participant shared:

‘I was uninformed about my child’s postoperative pain and was expecting the nurses to give me much information.’ (Biological mother, 25 years)

**Expectations from the hospital:** The caregivers expected the children after surgery to be put in a different ward but they were put in the same ward with adults. The caregivers shared:

‘After surgery, I was expecting my child and I to be sent to the children’s ward but we were brought to an adult ward which was not comfortable.’ (Biological mother, 50 years)

‘I thought my child would be sent to the children’s ward after the surgery but that was not the case.’ (Biological mother, 30 years)

## Discussion

### Theme one: Caregiver’s experiences of caring for children with postoperative pain

Caregivers in this study confirmed that their children’s postoperative pain had a significant emotional impact on them. These emotional impacts stem from anxiety, sorrow and feeling financially burdened. This study found that caregivers experienced anxiety when their children experienced postoperative pain. Similarly, Yayan and colleagues in their study found that caregivers had high anxiety levels when their children were in pain (Yayan et al. 2020). On the other hand, the caregiver’s anxiety also affects the child’s reaction to the stress of the surgery, both physically and emotionally (Getahun et al. 2020). Caregivers of children undergoing surgery experience sorrow, which can lead to chronic sorrow if not managed well. A review conducted by Coughlin and Sethares (2017) found that among the caregivers, mothers experienced more sorrow. Caregivers’ psychological support is crucial for managing children’s postoperative pain, as anxiety and sorrow are significant in this process. The study highlights the financial burden caregivers face because of postoperative pain in children, which significantly impacts their psychological experiences. A study conducted in India reported that the financial burden of caregivers of children undergoing surgery was high, emanating from direct and indirect costs (Mathias 2022). Similarly, another study added that the financial burden was high, especially in low-income economies (Platt et al. 2021).

### Theme two: Caregivers’ assessment of the postoperative pain of their children

This study found subjective, behavioural and physiological measures used by caregivers to assess their children’s postoperative pain. Regarding the subjective measures, a caregiver mentioned that a 3-year-old child could verbalise his pain. In the same vein, Herr et al. (2019) assert that children as young as 3 years can verbalise pain; however, it is not until 8 years that the subjective measure of assessing pain can be reliably used. On the contrary, Beltramini and colleagues believe that subjective measures should be used for children 6 years and above (Beltramini, Milojevic & Pateron 2017). There exists a significant correlation between age and the level of development, which in turn impacts the capacity of children to comprehend and articulate pain (Bakir et al. 2021). More research is needed to ascertain the exact age at which children can verbalise their pain, especially in the caregivers’ presence.

The caregivers in this study utilised behavioural measures by caregivers to evaluate their children’s postoperative pain, including crying, facial expression, irritability, restlessness, pointing to the wound, difficulty lying and food refusal. To the caregivers, their children cried when they were in pain, which was different from the usual crying of the child. Similarly, Carollo et al. (2023) pointed out that the child’s cry is diverse in various circumstances and requires a caregiver in constant touch with the child to differentiate. Additionally, most pain assessment scales employ crying as part of the assessment dimensions. Caregivers used facial expressions to assess children’s postoperative pain, describing their faces as squeezing, frowning, unusual and unhappy. Craig, Prkachin and Grunau (2011) assert that humans display pain by lowering their eyebrows, squeezing their eyes, wrinkling their noses, lifting their upper lips and opening their mouths. Children use facial expressions to express their feelings, requiring caregivers to respond to these cues promptly. However, Hughes, Chivers and Hoti (2023) caution that a thin line exists between pain-related facial expressions and other emotions. Concerning this, caregivers should be very observant in distinguishing pain-related facial expressions from other emotions. Children experiencing pain show irritable behaviour, such as restlessness, which caregivers use for pain assessment. Irritability has been reported in the literature as a symptom of possible pain (Nelson et al. 2018). Caregivers should identify postoperative pain as the principal cause of irritable behaviour using children’s gestures like pointing and touching the wound to detect their pain. Babaei et al. (2018) supported this assertion in their research by reporting that preverbal children from 12 months old use pointing to communicate. However, there is scarce literature on using these pointing and touching gestures to assess the postoperative pain of children.

Furthermore, the inability of the child to lie on the wound site was used by caregivers in this study to indicate postoperative pain. Again, concerning the behavioural measures of assessing pain, some of the caregivers mentioned that when their children were experiencing postoperative pain, they refused food.

Similarly, a study conducted by Ofosu Dwamena et al. revealed that children with postoperative pain refused food (Ofosu Dwamena et al. 2020). The caregivers in this study used increased body temperature as the only physiological measure for assessing postoperative pain. Though increased temperature is associated with infection (Walter et al. 2016), there is a scarcity of literature on postoperative pain increasing the body temperature of children; hence, much research is needed in this area.

### Theme three: Caregivers’ assistance with management of the postoperative pain of their children

Treatment of postoperative pain in literature involves the use of both pharmacological and non-pharmacological methods. However, caregivers in this study employed only non-pharmacological measures to assist in the management of the children’s postoperative pain. Psychosocial support given by the caregivers emanated as one of the non-pharmacological strategies that were utilised. The specific psychosocial interventions identified were caregivers’ presence, reassurance and walking while carrying the child. The caregivers’ presence is significant in caring for the hospitalised child (Azak, Aksucu & Çağlar 2022). During hospitalisation, nurses should support the caregiver in participating in the care (Sundal & Vatne 2020), not just being present. The caregivers also used reassurance to treat the postoperative pain of their children. This is consistent with a study by McMurtry et al. (2007) stating that the caregiver’s reassurance is helpful for the child to deal with stressful situations. Walking while carrying the child was used by the caregivers in this recent study as a psychosocial method for managing the postoperative pain of children. However, there are limited studies on walking while carrying the child; hence, research is needed in this area. Various distraction methods were used in this study to manage the postoperative pain of children. These methods included touch, music, video watching and conversation. Touch was used in this current study to distract children from postoperative pain. Similarly, Rezai and colleagues reported on using touch as a distraction method (Rezai et al. 2017). The caregivers in this study used music to treat their children’s postoperative pain. This finding is consistent with a study conducted in Iran (Shahrbabaki et al. 2023). The use of video was also identified as another distraction method. The video was in the form of animated cartoons. Similarly, video has been placed in literature as helpful in managing the postoperative pain of children (Luengo et al. 2023; Rezai et al. 2017). Conversation is also vital in distracting children’s postoperative pain, as recommended by best practice guidelines (American Academy of Pediatric Dentistry 2022; Wigg & Elson 2022). Lastly, breastfeeding was also utilised by caregivers to manage the postoperative pain of children.

Similarly, a previous study reported that breastfeeding helped manage the postoperative pain of children (Koukou et al. 2022). The same study added that direct breastfeeding was more beneficial than feeding the child with expressed breast milk using a cup or spoon. The caregivers in this study expected the nurses to give their children analgesics whenever there was pain. This finding is contrary to a study conducted in America that reported that a significant proportion of the caregivers used in the study (64%) expressed concern regarding the potential side effects associated with administering pain medication to children (Rosales et al. 2016). Also, in another study, 49.2% of the caregivers held the belief that pain medications should be minimised in children because of the possible side effects (Valizadeh, Ahmadi & Zarea 2016). The contradictory findings in this study could be attributed to the caregivers’ inadequate knowledge of pharmacological interventions and their side effects.

### Theme four: Expectations of caregivers concerning the management of their children’s postoperative pain

Again, the caregivers in this study expected the nurses to be present with the child while managing postoperative pain. This finding could be attributed to nurses and caregivers not interacting well during children’s pain management. Additionally, the caregivers expected to receive adequate information, but that was not the case. A study in Guatemala found that when caregivers were provided with sufficient information concerning their children’s postoperative pain using pictorial illustrations, it helped them to retain the information (Card et al. 2023). The caregivers in this study also expected the children to be nursed in the children’s ward after surgery and not an adult ward. It appeared that it increased the workload, which seemed to hinder effective pain management (Ofosu et al. 2021).

## Limitations

The study was limited to the caregivers in a regional hospital who were purposively sampled. Therefore, it is important to note that the findings of this study cannot be generalised to the entire region. Though the findings are not generalisable, the study can provide important information about caregiver’s contribution to the management of postoperative pain among children (0–3 years).

## Recommendations

Healthcare providers should actively involve the caregivers from the beginning during hospitalisation and educate them on the process that follows in the management of postoperative pain of their children. Also, healthcare providers should be clear on the role of the caregiver by providing education on non-pharmacological measures such as breast milk, the use of touch, video and conversation that can be used by the caregiver to assist in managing the children’s postoperative pain. Healthcare providers should evaluate the financial situation of caregivers whose children are undergoing surgery to identify suitable financial support systems.

## Conclusion

This study focused on caregivers’ experiences with hospitalised children’s postoperative pain, caregiver’s assessment and assistance with the management of postoperative pain of their children, and expectations of caregivers concerning the management of their hospitalised children’s postoperative pain. Caregivers in this study acknowledged the fact that the postoperative pain experienced by their children had a noteworthy emotional effect on them. The caregivers also assisted in the management of their hospitalised children’s postoperative pain with measures such as being present with the child, reassurance and breastfeeding. In summary, the findings of this study highlight the need for caregivers to be given adequate education on their role in the management of postoperative pain of their hospitalised children (0–3 years).
